# Phenotypic and Genomic Insights into *Schleiferilactobacillus harbinensis* WU01, a Candidate Probiotic with Broad-Spectrum Antimicrobial Activity Against ESKAPE (*Enterococcus faecium*, *Staphylococcus aureus*, *Klebsiella pneumoniae*, *Acinetobacter baumannii*, *Pseudomonas aeruginosa*, and *Enterobacter*) Pathogens

**DOI:** 10.3390/foods14071161

**Published:** 2025-03-27

**Authors:** Chonticha Romyasamit, Komwit Surachat, Nawanwat C. Pattaranggoon, Pinkanok Suksabay, Uttapol Permpoon, Tae-Gyu Nam, Phoomjai Sornsenee

**Affiliations:** 1 Department of Medical Technology, School of Allied Health Sciences, Walailak University, Nakhon Si Thammarat 80160, Thailand; chonticha.ro@wu.ac.th (C.R.); pinkanok.su@mail.wu.ac.th (P.S.); 2 Research Center in Tropical Pathobiology, Walailak University, Thasala District, Nakhon Si Thammarat 80160, Thailand; 3 Center of Excellence in Innovation of Essential Oil and Bioactive Compounds, Walailak University, Nakhon Si Thammarat 80160, Thailand; 4 Department of Biomedical Sciences and Biomedical Engineering, Faculty of Medicine, Prince of Songkla University, Hat Yai, Songkhla 90110, Thailand; komwit.s@psu.ac.th; 5 Faculty of Medical Technology, Rangsit University, Muang Pathumthani, Pathumthani 12000, Thailand; nawanwat.p@rsu.ac.th; 6 Department of Pharmacy, Institute of Pharmaceutical Science and Technology, Hanyang University ERICA, Ansan 15588, Republic of Korea; uttapol.pem@gmail.com (U.P.); tnam@hanyang.ac.kr (T.-G.N.); 7 Department of Family and Preventive Medicine, Faculty of Medicine, Prince of Songkla University, Songkhla 90110, Thailand

**Keywords:** *Schleiferilactobacillus harbinensis*, probiotics, antimicrobial activity, ESKAPE pathogens, whole-genome sequencing

## Abstract

The increasing prevalence of multidrug-resistant (MDR) pathogens, particularly ESKAPE bacteria, necessitates alternative antimicrobial strategies. Probiotics, particularly lactic acid bacteria, protect against pathogenic infections. This study aimed to characterize *Schleiferilactobacillus harbinensis* WU01, isolated from fermented palm sap, and evaluate its probiotic potential and antimicrobial activity. Its probiotic characteristics were assessed based on low-pH and bile tolerance, auto-aggregation, hydrophobicity, and adhesion to Caco-2 cells. Antimicrobial activity against ESKAPE pathogens was evaluated using the agar well diffusion assay. Whole-genome sequencing (WGS) and in silico analysis were performed to identify bacteriocin-related genes, virulence factors, and antibiotic-resistance genes. WU01 exhibited a strong tolerance to gastrointestinal conditions, with high survival rates under acidic and bile-salt environments. *S. harbinensis* WU01 demonstrated significant auto-aggregation, high hydrophobicity, and strong adhesion to Caco-2 cells. Antimicrobial assays revealed inhibitory activity against MDR ESKAPE pathogens, which correlated with the presence of bacteriocin-related genes, including those homologous to Carnocin_CP52. Molecular dynamics (MDs) simulations confirmed the interaction of Carnocin_CP52 with bacterial membranes, suggesting a mechanism for pathogen disruption. WGS confirmed the absence of virulence and antimicrobial-resistance genes, confirming its safety for probiotic applications. These findings suggest that *S. harbinensis* WU01 possesses probiotic properties and antimicrobial activity against ESKAPE pathogens. The combined results highlight its potential application in functional foods and therapeutic interventions.

## 1. Introduction

The increasing prevalence of antimicrobial resistance, largely driven by the overuse and misuse of antibiotics, has become a critical global health concern. The World Health Organization has designated the ESKAPE group of six nosocomial pathogens (*Enterococcus faecium*, *Staphylococcus aureus*, *Klebsiella pneumoniae*, *Acinetobacter baumannii*, *Pseudomonas aeruginosa*, and *Enterobacter* spp.) as high-priority pathogens owing to their significant multidrug-resistant (MDR) and extensively drug-resistant characteristics [1,2]. These bacteria possess several common biological traits, including their ability to adapt to modern healthcare environments, adopt diverse mechanisms to acquire resistance genes, and spread globally through the proliferation of high-risk clones [3]. To address this threatening challenge, identifying and developing novel therapeutic agents, including probiotics, are essential as potential alternatives to conventional antibiotics.

Probiotics are live microorganisms that confer a health benefit on the host when administered substantially [4]. Probiotics are a diverse group of beneficial microorganisms, including bacteria and yeasts. They confer health benefits to the host when consumed in adequate quantities. Some of the most well-known probiotic genera include Bifidobacterium, Bacillus, *Saccharomyces boulardii*, and lactic acid bacteria (LAB), widely recognized for their role in gut health, immune modulation, and antimicrobial activity [5]. Many of these microorganisms have been categorized as “Generally Recognized as Safe (GRAS)”, a designation that has significantly contributed to the rapid growth and innovation within the food industry [6]. Previous research has shown that probiotics offer several benefits, including enhancing the immune system, alleviating symptoms of irritable bowel disease, preventing and managing diarrhea and gastrointestinal disease, reducing allergy severity, and exhibiting significant anti-inflammatory, anti-cancer, and antimicrobial properties [7,8].

LAB, including *Lactobacillus*, *Leuconostoc*, *Pediococcus*, *Lactococcus*, *Streptococcus*, and *Schleiferilactobacillus harbinensis* (formerly *Lactobacillus harbinensis*), belong to the normal microbiota of the human and animal mucosa [9]. There are Gram-positive, non-spore-forming, facultatively heterofermentative, catalase-negative microorganisms that are essential to the food and feed industries [10]. They are traditionally characterized by their ability to produce lactic acid as the primary end-product of carbohydrate metabolism, a trait that underscores their critical role in fermentation processes and food preservation [10,11].

*Schleiferilactobacillus harbinensis* (formerly *Lactobacillus harbinensis* sp. nov.) was initially isolated from traditional fermented vegetables (Suan cai) in China [12] and subsequently from dairy systems [13], silage [14], and fermented tofu whey. This strain has a genome size of 3.14 Mbp and a DNA G+C content of 53–54 mol% [12]. Previous research has shown that *S. harbinensis* M1, supported by complete genome sequencing, exhibits a strong acid-producing capacity and significant proteolytic activity. Additionally, the strain enhances antioxidant and antiproliferative properties [9]. It produces various postbiotics, such as exopolysaccharides. The exopolysaccharide F-EPS1A from *S. harbinensis* Z171 remains stable during digestion, promotes beneficial gut bacteria, reduces harmful microbes, reduces cholesterol, exerts antioxidant and anti-hypoglycemic effects, and increases short-chain fatty acids (such as butyric acid) [15,16]. Fermented palm sap is a naturally fermented beverage obtained from the sap of the palmyra palm (*Borassus flabellifer Linn.*), commonly consumed in tropical regions, particularly in southern Thailand. In our previous study, 10 *Lactobacillus* strains, including *L. paracasei* (8/10), *L. fermentum* (1/10), and *L. brevis* (1/10), were isolated and identified as potential probiotics [17].

Therefore, this study aimed to evaluate the potential probiotic properties of *S. harbinensis* WU01 isolated from fermented palm sap against ESKAPE pathogens, sequencing the genome of *S. harbinensis* WU01 and evaluating its safety profile.

## 2. Materials and Methods

### 2.1. Bacterial Strains and Culture Conditions

The 12 reference strains, including foodborne pathogens and hospital-associated pathogens, were obtained from the Department of Medical Sciences, Thailand (DMST): *Enterococcus faecium*, *Enterococcus faecalis* DMST 4736, *Staphylococcus aureus* subsp. *aureus* ATCC 6538, methicillin-resistant *Staphylococcus aureus*, *Klebsiella pneumoniae*, *Acinetobacter baumannii*, *Pseudomonas aeruginosa*, *Enterobacter* spp., *Escherichia coli* DMST 4212, *Salmonella typhi* DMST 22842, *Salmonella enteritidis* DMST 15676, and *Shigella flexneri* DMST 44237.

*S. harbinensis* WU01 was isolated from fermented palm sap and identified through Matrix-Assisted Laser Desorption/Ionization Time-of-Flight Mass Spectrometry (MALDI-TOF MS) and WGS. All strains were cultured on a trypticase soy agar (TSA; HiMedia, Mumbai, India) and incubated for 24 h at 37 °C under aerobic conditions. Colonies were subsequently transferred to a trypticase soy broth (TSB; HiMedia, Mumbai, India) and incubated for 18 h at 37 °C. Each strain was preserved at −80 °C in TSB supplemented with 30% glycerol (Sigma-Aldrich, St. Louis, MO, USA) until further testing.

### 2.2. Characterization of Probiotic Properties

#### 2.2.1. Screening of Antipathogenic Activity

The antimicrobial activity of *S. harbinensis* WU01 against the 12 pathogenic microorganisms was evaluated using the agar well diffusion assay, as described by Nigam, Kumar, Iyengar, and Bhola [18]. The inhibition zones observed around the wells were measured to assess the antimicrobial efficacy of *S. harbinensis* WU01. Additionally, the effects of the cell-free supernatant (CFS) of *S. harbinensis* WU01 on the morphology of the pathogens were investigated using scanning electron microscopy (SEM). A SEM sample was prepared following the antipathogenic activity assay. Bacterial samples were centrifuged at 5000× *g* at 5 min, placed on slides, fixed with glutaraldehyde, and dehydrated using an ethanol gradient. They were subjected to drying at the critical point, mounted on stubs, and gold-coated before visualization.

#### 2.2.2. Acid Tolerance

The acid tolerance of the probiotic (*S. harbinensis*) at pH 2.0, 3.0, and 6.5 was assessed following the method of Sornsenee, et al. [17]. Briefly, *S. harbinensis* WU01 cells were harvested through centrifugation (8000× *g*), and the pellet was washed with phosphate-buffered saline (PBS). Subsequently, the cell suspension (10^8^ CFU/mL) was adjusted to pH 2.0 or 3.0 using hydrochloric acid and incubated for 3 h at 37 °C. Next, cell viability was determined by plating the treated cells on an De Man–Rogosa–Sharpe (MRS) agar (HiMedia, Mumbai, India), and the survival rate (%) was calculated using Equation (1):

Survival rate (%) = (Final (Log CFU/mL)/Initial (Log CFU/mL)) × 100(1)

#### 2.2.3. Pepsin, Pancreatin, and Bile-Salt Tolerances

The digestive tolerance of *S. harbinensis* WU01 to pepsin, pancreatin, and bile salts was evaluated following a previously described method [17]. Briefly, pepsin (3 g/L), pancreatin (1 g/L), and 0.3% (*w*/*v*) bile-salt solutions (Sigma-Aldrich) were prepared in an MRS broth adjusted to pH 2.0, 8.0, and 8.0, respectively. Overnight bacterial cultures were harvested through centrifugation, and the resulting pellet was washed and resuspended in an MRS broth containing the respective pepsin, pancreatin, or bile-salt solutions. The cell suspensions (10^8^ CFU/mL) were incubated for 3 (pepsin) or 4 (pancreatin and bile salts) h at 37 °C. Following incubation, the viability of *S. harbinensis* WU01 was determined through plating on an MRS agar, and the survival rate (%) was calculated using Equation (1).

#### 2.2.4. Auto-Aggregation

The auto-aggregation ability of LAB isolates was evaluated following a previously described method [17] with minor modifications. Briefly, overnight cultures of *S. harbinensis* WU01 were harvested through centrifugation at 8000× *g* for 5 min and washed twice with PBS. Subsequently, the pellet was resuspended in PBS at pH 7.2. The suspensions were incubated anaerobically at 37 °C, and the absorbance of the upper suspension was measured at 600 nm at time intervals of 0, 2, 4, and 24 h. The auto-aggregation percentage was calculated using the following formula:

Auto-aggregation % = [1 − (A_time_/A_0_) × 100], (2)

where A_time_ is the absorbance at a particular time, and A_0_ is the absorbance at time 0.

#### 2.2.5. Cell-Surface Hydrophobicity

The hydrophobicity of *S. harbinensis* WU01 was assessed using a xylene extraction method, as previously described [17]. Briefly, *S. harbinensis* WU01 cells from an overnight culture were harvested through centrifugation, washed, and resuspended in PBS. The optical density (OD) of the suspension was measured at 600 nm (OD_600_). Subsequently, xylene was added to the cell suspension, and the mixture was incubated for 30 min at 37 °C without shaking, allowing the separation of aqueous and organic phases. The OD_600_ of the aqueous phase was subsequently measured, and the hydrophobicity percentage (H%) was calculated using Equation (3).

H% = [(A0 − A)/A0] × 100, (3)

where A0 and A are the absorbances measured pre- and post-xylene extraction, respectively.

#### 2.2.6. Adhesion to Human Intestinal Epithelial Cells

The adhesive ability of *S. harbinensis* WU01 in terms of adherence to human epithelial intestinal Caco-2 cells was assessed, as described by Sornsenee et al. [17]. Briefly, Caco-2 cells were cultured in Dulbecco’s modified Eagle’s medium (Thermo Fisher Scientific, Waltham, MA, USA) with supplements until an 80% confluence was achieved and co-cultured with 1 × 10^8^ CFU/mL *S. harbinensis* WU01 for 2 h. Subsequently, adherent bacteria were plated on an MRS agar, and the adhesion percentage was calculated using Equation (4):

% adhesion ability = (V1 × 100)/V0 (4)

where V0 is the initial viable count, and V1 is the viable count adhered to the Caco-2 cells after incubation.

#### 2.2.7. Scanning Electron Microscopy (SEM)

The method described by Sornsenee et al. (2021) [17] was followed to prepare Caco-2 cells for SEM. Untreated Caco-2 cells and cells treated with *S. harbinensis* WU01 were fixed on coverslips using 2.5% (*v*/*v*) glutaraldehyde (Sigma-Aldrich) for 24 h at 4 °C. The fixed cells were dehydrated through sequential incubations with ethanol solutions of increasing concentrations (40%, 60%, 80%, and 95% *v*/*v*) for 15 min each, followed by two 15 min incubations with 100% ethanol (Thermo Fisher Scientific, Waltham, MA, USA). Subsequently, the coverslips were air-dried at 25 °C for 30 min, mounted on stubs, and coated with gold for 3 min. The samples were subsequently visualized using a field-emission scanning electron microscope (Oxford Instruments, Quanta, FEG 250, Tokyo, Japan).

#### 2.2.8. Susceptibility to Antibiotics

Antibiotic susceptibility was assessed following the guidelines of the Laboratory Standards Institute (2024) [19]. The selected antibiotics (Oxoid, Hampshire, UK) included ampicillin (10 µg), vancomycin (30 µg), gentamicin (10 µg), erythromycin (15 µg), clindamycin (2 µg), tetracycline (30 µg), kanamycin (30 µg), chloramphenicol (30 µg), and streptomycin (10 µg).

#### 2.2.9. Hemolytic Test

The hemolytic activity of the LAB isolates was evaluated using blood agar (HiMedia, Mumbai, India), following the method described by Leite et al. [20]. The presence of β-hemolysis is characterized by the formation of clear zones surrounding the bacterial colonies; α-hemolysis is indicated by greenish discoloration in the surrounding medium.

### 2.3. Characterization of the Active Antimicrobial Substances of S. harbinensis WU01

#### 2.3.1. Bacteriocin Screening

The production of antimicrobial substances, including bacteriocins, by *S. harbinensis* WU01 was assessed following the method of Sornsenee et al. (2021) [17]. Overnight LAB cultures were centrifuged (7000× *g*, 10 min), and the supernatants were adjusted to a pH of 6.5 with 1 N NaOH. After treatment with 1 mg/mL proteinase K (Sigma-Aldrich) at 30 °C for 2 h, the enzyme was heat-inactivated at 80 °C for 10 min. The filtered supernatants (0.2-μm) were put on MRS agar plates pre-inoculated with pathogenic bacteria and incubated under aerobic conditions at 37 °C for 48 h.

#### 2.3.2. Hydrogen Peroxide (H_2_O_2_) Production and Bile Salt Hydrolase (BSH) Activity

H_2_O_2_ production by selected isolates was tested following the method described by Song et al. [21]. In brief, MRS agar was supplemented with 0.25 mg/mL tetramethylbenzidine (Sigma-Aldrich) and 0.01 mg/mL horseradish peroxidase (Sigma-Aldrich). The inoculated plates were incubated under anaerobic conditions at 37 °C for 48 h. H_2_O_2_ production was evaluated based on the blue color development, which was considered positive.

BSH activity was measured following the method described by Wang et al. [22]. In brief, overnight cultures were streaked onto MRS agar supplemented with 0.5% (*w*/*v*) taurodeoxycholic acid (Sigma-Aldrich) and 0.035% (*w*/*v*) CaCl_2_ (Sigma-Aldrich). The plates were incubated under anaerobic conditions at 37 °C for 2 days. BSH activity was identified by the presence of opaque halos around the colonies, indicating the precipitation of deconjugated bile acids.

### 2.4. Genomic Analysis

#### 2.4.1. DNA Isolation and WGS

The genomic DNA of *S. harbinensis* WU01 was extracted and purified using a DNeasy extraction kit (QIAGEN, Hilden, Germany) according to the manufacturer’s protocol. Briefly, bacterial cells were lysed in 180 µL of lysis buffer for 30 min at 37 °C, followed by incubation with 25 µL of proteinase K and 200 µL of buffer AL for 30 min at 56 °C. Ethanol (200 µL) was added, and the mixture was centrifuged, washed with buffer AW2, and eluted in buffer AE. DNA purity was assessed using spectrophotometry (A260/A280) and confirmed using agarose gel electrophoresis.

The DNA sample was sent to the Beijing Genomics Institute for WGS using 150 bp paired-end reads on the MGISEQ-2000 platform. The sequencing reads were assembled and annotated using the BacSeq v1.0 pipeline [23], which integrates multiple bioinformatics tools, including SPAdes [24], Prokka [25,26], QUAST [25,26], and BUSCO [27], for genome assembly, annotation, and quality assessment. Mobile genetic elements, prophages, and antimicrobial-resistance genes (ARGs) were analyzed using mobileOG-db [28], Phigaro [29], VirulenceFinder [30], and ResFinder web-based tools [31], respectively, with the ARG identification criteria set at 90% identity and a minimum length of 60%. CRISPR arrays and Cas proteins were identified using CRISPRCasFinder [32], while ribosomally synthesized and post-translationally modified peptides and bacteriocin-encoding genes were detected through sequence similarity searches using BAGEL4 [33].

#### 2.4.2. Pangenome Analysis and Comparative Genomics

The Roary pipeline [34] was used to analyze the pan-genome of *S. harbinensis* WU01 using a 95% BLASTp threshold and standard parameters to classify core, accessory, and unique protein families. Multiple gene alignments and phylogenetic trees were constructed using Geneious (Version 2025.0) [35] with the neighbor-joining method, and bootstrap testing with 500 repetitions was performed to evaluate tree reliability. Comparative genome analysis was conducted using Proksee 1.0.0a6 [36] and BLASTn [37] to assess coding sequence similarities, while average nucleotide identity (ANI) was calculated using OrthoANI [38].

### 2.5. Computational Methods

#### 2.5.1. System Preparation

The amino acid sequence of Carnocin_CP52 (see Supporting Information) was submitted to the UniProt database (UniProtKB code: A0A5P8M1F1) to confirm that it corresponds to a bacteriocin immunity protein produced by *Schleiferilactobacillus harbinensis*. Since the 3D structure was not available in the Protein Data Bank (PDB), the structure predicted by AlphaFold [39] was used as the starting model. The protonation states of all ionizable amino acids were assigned at pH 7.4 using the APBS web service. The CHARMM-GUI Membrane Builder [40] was utilized to construct the protein–membrane systems. The simplified lipid bilayer consisted of 80% phosphatidylcholine (DMPC) and 20% phosphatidylglycerol (DOPG) [41]. Both simplified lipid bilayers were symmetric in terms of the lipid composition of the upper and lower leaflets, with each leaflet consisting of 124 DMPC and 31 DOPG molecules. The structure of Carnocin_CP52 was initially positioned approximately 15 Å above the upper leaflet of the bilayers (see Figure 4A). The system was then solvated using the TIP3P water model. To neutralize the system and achieve a final NaCl concentration of 0.15 M, 120 sodium ions and 56 chloride ions were added as counterions.

#### 2.5.2. Molecular Dynamics Simulations

Atomistic molecular dynamics simulations were carried out to study the mode of interactions of Carnocin_CP52 with a model lipid bilayer. The system was parameterized using the AMBER force fields, specifically ff19SB for proteins, Lipid21 for lipids, GAFF2 for small molecules, and the 12-6-4 ion parameters for ions. Water molecules were explicitly modeled using the TIP3P water model. All simulations were performed using the AMBER molecular dynamics package (AMBER22) [42]. The system was first minimized using a two-step process. The first step involved an initial minimization with constraints on the solute to allow relaxation of the solvent molecules. This was followed by full minimization of the entire system without constraints. After minimization, equilibration was conducted in multiple stages under the NVT and NPT ensembles. For the NVT equilibration, the temperature of each simulated system was gradually increased to 310 K over 200 ps using Langevin dynamics with a collision frequency of 1.0 ps^−1^. A harmonic positional restraint of 100 kcal/mol·Å^2^ was applied to the Cα atoms of the protein. Additionally, restraints were applied to membrane phosphate atoms (P31) of phosphatidylglycerol (PGR) and phosphatidylcholine (PC) lipids, with a force constant of 2.5 kcal/mol·Å^2^. Following NVT equilibration, the system was subjected to NPT equilibration to adjust the pressure and density. The system was maintained at 1 atm of pressure using a Berendsen barostat. A nonbonded cutoff of 12.0 Å was applied to account for long-range interactions. Production simulations were then conducted under NPT conditions without restraints until 500 ns was reached.

### 2.6. Statistical Analyses

All experiments were performed in triplicate. Results were presented as the mean ± standard deviation. Statistical analysis was performed using a one-way analysis of variance in GraphPad Prism 5 (GraphPad Software, San Diego, CA, USA), with significance set at *p* < 0.05.

## 3. Results

### 3.1. Bacterial Isolation and Identification

*Schleiferilactobacillus harbinensis* WU01 was isolated from fermented palm sap collected from local markets in Songkhla Province, Thailand. Initial species identification was performed using Matrix-Assisted Laser Desorption/Ionization Time-of-Flight Mass Spectrometry, yielding a score of >2.0, indicating high-confidence identification. Confirmation was further validated through short-read genomic DNA sequencing using the MGISEQ 2000 platform, and we identified *S. harbinensis* WU01 using FastANI, which calculates the average nucleotide identity (ANI) between our strain and a reference genome. For comparison, we used the genome of *S. harbinensis* NSMJ42, obtained from the NCBI database. The ANI value between *S. harbinensis* WU01 and the reference strain was 97.96%, exceeding the 95% species demarcation threshold. This high sequence similarity confirms that our strain belongs to *S. harbinensis*.

### 3.2. Characterization of Probiotic Properties

#### 3.2.1. Stimulation of Gastrointestinal Tract (GIT) Tolerance

**Figure 1:**
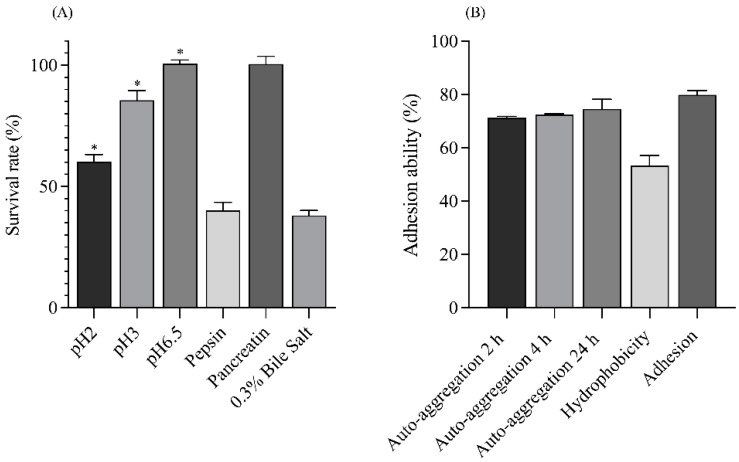
Probiotic properties of *S. harbinensis* WU01. (**A**) Survival rates of *Schleiferilactobacillus harbinensis* WU01 under various gastric and intestinal conditions (pH tolerance and pepsin, pancreatin, and bile-salt exposure). (**B**) Percentage of auto-aggregation (2 h, 4 h, and 24 h), hydrophobicity, and cell adhesion of *S. harbinensis* WU01 to Caco-2 cells. Statistical analysis was conducted for pH tolerance (pH 2.0, pH 3.0, and pH 6.5) and auto-aggregation across different time points using one-way ANOVA, with significant differences indicated by *p* < 0.05 (*).

The GIT tolerance properties of *S. harbinensis* WU01 were evaluated (Figure 1A). The strain exhibited high acid tolerance and survivability, with survival rates of 100.60 ± 1.55%, 85.59 ± 4.03%, and 60.28 ± 2.88% at pH 6.5, 3, and 2 after 3 h of incubation, respectively. *Schleiferilactobacillus harbinensis* WU01 demonstrated strong tolerance to pancreatic enzyme treatment at pH 8.0, with a survival rate of 100.48 ± 3.20%, and moderate tolerance to pepsin at pH 2, with a survival rate of 40.11 ± 3.37%. Additionally, approximately 38.16 ± 1.97% of the cells survived in the presence of 0.3% bile salts. These results confirm the ability of *S. harbinensis* WU01 to tolerate GIT conditions, highlighting its probiotic potential.

#### 3.2.2. Auto-Aggregation and Enhancement of Adhesion Ability

**Figure 2:**
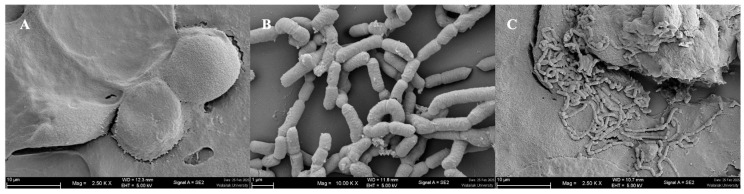
Scanning electron microscopy (SEM) of Caco-2 cells where the *S. harbinensis* WU01 adhered to the surface of Caco-2 cells: (**A**) untreated Caco-2 cells as control. (**B**) *S. harbinensis* WU01; (**C**) *S. harbinensis* WU01 adhered to the surface of Caco-2 cells.

The auto-aggregation percentages of *S. harbinensis* WU01 were 71.40 ± 0.43%, 72.51 ± 0.27%, and 74.65 ± 3.53% after 2, 4, and 24 h at 37 °C, respectively (Figure 1B). Additionally, *S. harbinensis* WU01 exhibited high hydrophobicity at 53.29 ± 3.83% and strong adhesion to Caco-2 cells, with an adherence rate of 79.88 ± 1.68%. SEM confirmed the tight interaction between *S. harbinensis* WU01 and Caco-2 cells (Figure 2). These findings demonstrate that *S. harbinensis* WU01 possesses strong probiotic properties, including resilience under stressful GIT conditions and enhanced adherence to intestinal cells.

#### 3.2.3. Antibiotic Susceptibility and Hemolysis

*Schleiferilactobacillus harbinensis* WU01 exhibited susceptibility to certain antibiotics (ampicillin, erythromycin, clindamycin, tetracycline, and chloramphenicol). However, the strain demonstrated resistance to vancomycin, gentamicin, and kanamycin (Supplementary Table S1). Furthermore, no hemolytic activity was observed on the blood agar plates, indicating gamma-hemolysis and confirming their non-hemolytic nature.

### 3.3. Characterization of Antimicrobial Substances

#### 3.3.1. Antimicrobial Activity

*Schleiferilactobacillus harbinensis* WU01 cells strongly inhibited *Pseudomonas aeruginosa* with the highest inhibitory zone at 19.33 ± 0.58 mm, followed by *Staphylococcus aureus* subsp. *aureus* ATCC 6538 (19.33 ± 0.57 mm) and *E. faecalis* DMST 4736 (16.33 ± 0.58) (Table 1). Notably, *S. harbinensis* WU01 exhibited antimicrobial activity against ESKAPE pathogens and other tested pathogens.

#### 3.3.2. Production of Antimicrobial Substances

*Schleiferilactobacillus harbinensis* WU01 produced antimicrobial substances that inhibited pathogenic bacteria, as confirmed by agar well diffusion assays. The inhibitory activity remained unaffected by proteinase K treatment or pH neutralization, suggesting the presence of proteinaceous agents (bacteriocins, particularly those homologous to Carnocin_CP52), organic acids, and other proteinaceous compounds. Additionally, BSH activity was detected in *S. harbinensis* WU01, whereas H_2_O_2_ production was not observed.

#### 3.3.3. Genome Characteristics

**Figure 3:**
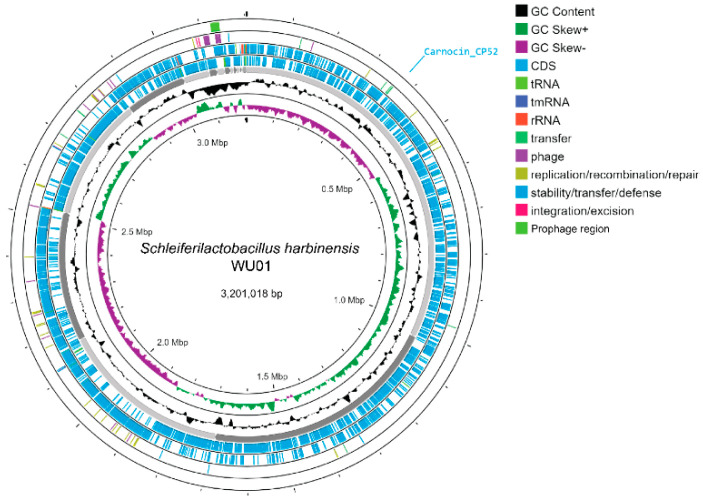
Circular genome maps of *S. harbinensis* WU01.

The draft genome of *S. harbinensis* WU01 was 3,201,018 bp in length, comprising 28 contigs with a GC content of 53.31% (Table 2, Figure 3). No plasmids were detected. The genome includes 2993 coding sequences, 4 rRNA genes, 63 tRNA genes, and 1 tmRNA. To confirm the taxonomic classification, ANI analysis was performed against *Schleiferilactobacillus* species from the National Center for Biotechnology Information database. The ANI values of *S. harbinensis* WU01 compared to those of reference strains from the database confirmed its identity as *S. harbinensis* based on similarities in genome size, GC content, and ANI metrics. A BLASTP search using the BAGEL4 database was conducted to identify bacteriocins encoded within the genome. The analysis revealed one protein in *S. harbinensis* WU01 with similarity to Carnocin_CP52 (Supplementary Figure S1).

#### 3.3.4. In Silico Safety Evaluation

The ABRICATE tool, which used data from the virulence factor database (VFDB, http://www.mgc.ac.cn/VFs/ (accessed on 22 January 2025)), revealed no virulence genes in *S. harbinensis* WU01. This finding reinforces its safety for probiotic applications and supports its classification as a non-pathogenic strain.

No ARGs were identified in the ResFinder database, further corroborating its safety. Antibiotic susceptibility testing confirmed that *S. harbinensis* WU01 is susceptible to several antibiotics, including ampicillin, erythromycin, clindamycin, tetracycline, and chloramphenicol. Moreover, an analysis using the MEGFinder database revealed four insertion sequences within the T0701 strain, which contained no encoded genes, suggesting a lack of transmission capability.

Similarly, the genomic analysis revealed the presence of one intact and two incomplete phages in *S. harbinensis* WU01, along with two prophage regions (Supplementary Table S2). These findings provide further insights into the genomic composition of the strain while emphasizing its safety profile for potential probiotic applications.

### 3.4. Molecular Dynamics Simulations

**Figure 4:**
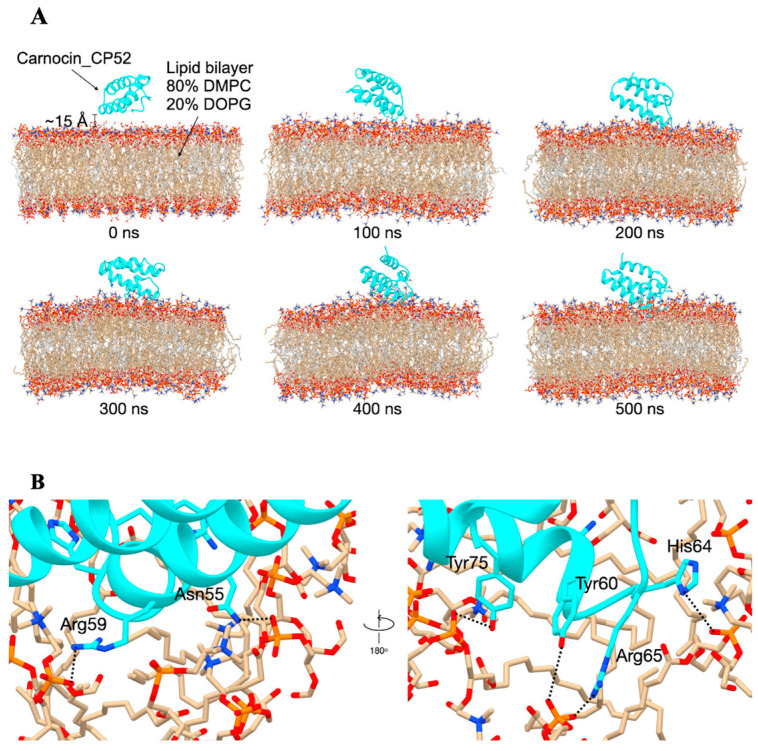
Molecular dynamics simulations. (**A**) Snapshots at critical simulation time points. The Carnocin_CP52 peptide is represented in cyan and was initially positioned approximately 15 Å above the upper leaflet of the bilayer. The lipid bilayer consists of 80% DMPC (tan) and 20% DOPG (gray). (**B**) The last snapshot (500 ns) represents the hydrogen bonds between Carnocin_CP52 (cyan color) and lipids. The key interacting residues are Asn55, Arg59, Tyr60, His64, Arg65, and Tyr75.

**Figure 5:**
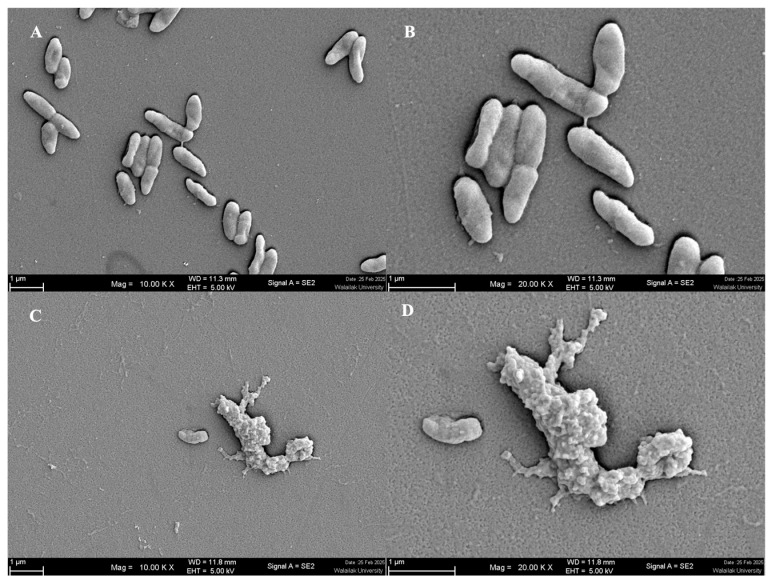
Morphology of *P. aeruginosa* (**A**,**B**) and *P. aeruginosa* after treatment with CFS of *S. harbinensis* WU01 (**C**,**D**) observed by SEM. Magnifications were revealed as (**A**,**C**) = 10,000×; (**B**,**D**) = 20,000×.

Snapshots at critical simulation time points reveal the interaction between the Carnocin_CP52 peptide (cyan) and the lipid bilayer (80% DMPC in tan, 20% DOPG in gray). Initially, the peptide is positioned approximately 15 Å above the membrane. By 100 ns, it moves closer to the bilayer, and by 200 ns, it begins interacting directly with the membrane surface. At later time points (300–500 ns), the peptide becomes more embedded, indicating interactions with the bilayer (Figure 4A). These simulation results align with experimental findings, where Carnocin_CP52 was observed to associate with the membrane and induce structural disruptions. The hydrogen bond interactions between Carnocin_CP52 and lipid molecules were analyzed from the 400–500 ns simulation using the CPPTRAJ module in AMBER22, with a distance cutoff of 3.0 Å and an angle cutoff of 120°. Key residues—Asn55, Arg59, Tyr60, His64, Arg65, and Tyr75—formed stable hydrogen bonds with lipid headgroups. Arg59 and Arg65 formed strong electrostatic interactions with lipid phosphate groups, with hydrogen bond occupancies of 77% and 56%, respectively. Tyr75 and Tyr60 engaged in hydrogen bonding via hydroxyl groups, with Tyr75 exhibiting an occupancy of 32%, while Asn55 interacted via ND2 with an occupancy of 47%, suggesting potential lipid selectivity. His64 also participated in hydrogen bonding interactions (occupancy 14%), contributing to the stabilization of the peptide–lipid interface (Figure 4A). These hydrogen bonding interactions highlight a stable electrostatic and polar network. The molecular dynamics simulation shows that the peptide associates with and perturbs the bilayer over time, potentially leading to membrane destabilization and causing membrane rupture. This result is supported by the morphological changes observed in *P. aeruginosa* treated with the CFS of *S. harbinensis* WU01, where bacterial cells exhibited abnormal shapes, including membrane disruption and breakdown, compared to the untreated control (Figure 5A–D).

## 4. Discussion

The increasing prevalence of infections caused by ESKAPE pathogens represents a critical global health concern. These pathogens significantly contribute to healthcare-associated infections and exhibit high levels of resistance to multiple antibiotics, complicating treatment strategies and leading to high morbidity and mortality [1,2]. The need for developing novel strategies to combat these pathogens is critical, and probiotics have emerged as a promising alternative. Probiotics are recognized for their ability to inhibit or eliminate pathogens, modulate the host immune system, and produce antimicrobial substances such as bacteriocins [6,43].

Fermented foods usually contain microorganisms with a Generally Recognized as Safe status, capable of producing various beneficial by-products and metabolites, including bacteriocins, ethanol, organic acids, fatty acids, and carbon dioxide [44]. In southern Thailand, particularly Songkhla Province, traditional fermented palm sap is a well-known and culturally significant beverage. Fermented palm sap contains diverse microbial populations, including genera such as *Lactococcus*, *Micrococcus*, *Streptococcus*, *Leuconostoc*, *Lactobacillus*, *Acetobacter*, and *Saccharomyces*. These microorganisms are crucial in generating metabolites that improve the flavor, texture, and health-promoting properties of the fermented product [17,45]. In this study, the *S. harbinensis* WU01 isolated from fermented palm sap had 3,201,018 bp and exhibited several key probiotic characteristics, including a low-pH, pepsin, pancreatin, and bile tolerance; strong adhesion to intestinal epithelial cells; and a high auto-aggregation capability [46]. Additionally, SEM provided direct visual evidence of *S. harbinensis* WU01 interacting with intestinal epithelial cells, confirming its potential for gut colonization and pathogen inhibition. These properties are essential for probiotics to survive gastrointestinal transit and colonize the intestinal tract. *S. harbinensis* WU01 showed high hydrophobicity, supporting its potential for epithelial adhesion. Moreover, bile salts represented some conditions of the small-intestine environment. The intestinal bile concentration is 0.3% and maintains food in the small intestine for 4 h [47].

The results are consistent with previous reports [48,49], which demonstrated that *L. paracasei* exhibits tolerance to gastrointestinal stress. *Lactobacillus paracasei* showed improved survival, particularly when associated with a food carrier, within the pH range of 3.0–5.0 in the gastrointestinal tract. Similarly, *Schleiferilactobacillus* cells displayed adaptability across a wide pH range, possibly owing to their ability to withstand environmental stress through physiological and biochemical modifications. These adaptations may include membrane restructuring, activation of H⁺-ATPase activity, and maintenance of cytoplasmic alkaline homeostasis via the production of metabolic enzymes and stress-related proteins [46,50].

The safety of potential probiotic strains for human consumption is a primary selection criterion. Probiotic bacteria may carry intrinsic or mobile genetic elements that confer antibiotic resistance, necessitating a comprehensive assessment of their antimicrobial susceptibility profiles to prevent potential clinical risks [51,52]. *Lactobacillus* species or *Schleiferilactobacillus* are inherently resistant to vancomycin and gentamicin and do not facilitate the horizontal transfer of antibiotic-resistance genes between isolates and species [53]. In this study, *S. harbinensis* WU01 exhibited no detectable ARGs and demonstrated an absence of hemolytic activity on blood agar plates. These findings are similar to those of previous studies, which have consistently demonstrated the safety and tolerability of *S. harbinensis* strains, supporting their potential as probiotic candidates.

Furthermore, *S. harbinensis* WU01 exhibited antimicrobial activity against ESKAPE pathogens and exhibited strong probiotic potential. Its antimicrobial properties were attributed to proteinaceous compounds, as evidenced by their stability following enzymatic degradation and pH neutralization. Genomic analysis revealed genes encoding bacteriocins, including those homologous to Carnocin_CP52, which are known for their potent antimicrobial activity against Gram-positive pathogens. Carnocin_CP52 exerts its antimicrobial effect by binding to the mannose phosphotransferase system on target bacteria, leading to pore formation, ion leakage, and cell death. Additionally, it may disrupt peptidoglycan synthesis and other essential cellular functions, enhancing its bactericidal activity [54]. *Lactobacillus casei* KACC92338 [55], *L. rhamnosus* SN21-1, and *L. plantarum* SN21-2 [56] harbor Carnocin_CP52, which exhibits antimicrobial activity against *Salmonella typhimurium*. Bacteriocins have been extensively studied for their ability to inhibit MDR bacteria while maintaining a favorable safety profile for probiotic applications [57]. Molecular docking could further elucidate the molecular interactions between bacteriocins produced by *S. harbinensis* WU01 and key pathogenic targets, such as *Pseudomonas aeruginosa*. These integrative methodologies deepen the understanding of bacteriocin-mediated inhibition and contribute to the development of targeted probiotic-based therapeutic strategies.

## 5. Conclusions

This study identified *S. harbinensis* WU01 as a promising probiotic strain with considerable antimicrobial activity against MDR pathogens, particularly ESKAPE bacteria. The strain demonstrated strong tolerance to gastrointestinal conditions, high adhesion to intestinal epithelial cells, and remarkable auto-aggregation, supporting its probiotic potential. Whole-genome sequencing confirmed the absence of virulence and antimicrobial-resistance genes, reinforcing its safety profile. The presence of bacteriocin-related genes, including those homologous to Carnocin_CP52, suggests a potential mechanism for its antimicrobial activity. Molecular dynamics simulations validated the interaction between Carnocin_CP52 and bacterial membranes, indicating its role in pathogen disruption. Thus, *S. harbinensis* WU01 is a strong candidate for functional food applications and antimicrobial interventions. Future in vivo studies and clinical trials are necessary to evaluate its efficacy and potential therapeutic applications.

## Figures and Tables

**Table 1:** Antimicrobial activity of the isolated *S. harbinensis* WU01 from fermented palm sap against 11 pathogens.

Bacteria	*S. harbinensis* WU01
*E. faecium*	++
*E. faecalis* DMST 4736	+++
*S. aureus* subsp. *aureus* ATCC 6538	+++
MRSA	++
*K. pneumoniae*	++
*A. baumannii*	++
*P. aeruginosa*	+++
*Enterobacter* spp.	ND
*E. coli* DMST4212	+++
*S. typhi* DMST 22842	+
*S. enteritidis* DMST 15676	+++
*S. flexneri* DMST 44237	++

ND, not detected (zone: <6 mm); +, inhibition zone 6–10 mm; ++, inhibition zone 11–15 mm; +++, inhibition zone > 16 mm; MRSA: methicillin-resistant *S. aureus.*

**Table 2:** Main genome features of *S. harbinensis* WU01.

Features	*S. harbinensis* WU01
Genome size (bp.)	3,201,018
Contigs	28
GC content (%)	53.31%
N50	453,387
L50	3
Number of CDS	2993
tRNA	63
rRNA	4
tmRNA	1
Bacteriocin-like encoding gene	1

## Data Availability

The original contributions presented in the study are included in the article/Supplementary Materials, further inquiries can be directed to the corresponding author.

## Supplementary Materials

The following supporting information can be downloaded at: https://www.mdpi.com/article/10.3390/foods14071161/s1, Figure S1: *Schleiferilactobacillus harbinensis* WU01 genome analysis of bacteriocin genes arrangement using the BAGEL4 The area of interest at Contig_1 indicates Carnocin_CP52; Figure S2: The plots of root-mean-square displacement (RMSD) of Cα atoms of Carnocin_CP52 peptide; Table S1: Antibiotic susceptibility of *Schleiferilactobacillus harbinensis* WU01; Table S2: Predicted prophage regions in the genome of *S. harbinensis* WU01; Table S3: The hydrogen bond interactions between Carnocin_CP52 and lipid molecules were analyzed from the 400–500 ns simulation.

## Abbreviations

The following abbreviations are used in this manuscript:

MDR	Multidrug resistant
LAB	Lactic acid bacteria
WGS	Whole-genome sequencing
SEM	Scanning electron microscopy
PBS	Phosphate-buffered saline
BSH	Bile Salt Hydrolase
ARGs	Antimicrobial-resistance genes
ANI	Average nucleotide identity
