# Supplementary material for: Phenotypic and Genomic Insights into Schleiferilactobacillus harbinensis WU01, a Candidate Probiotic with Broad-Spectrum Antimicrobial Activity Against ESKAPE (Enterococcus faecium, Staphylococcus aureus, Klebsiella pneumoniae, Acinetobacter baumannii, Pseudomonas aeruginosa, and Enterobacter) Pathogens
Source: Foods. 2025 Mar 27;14(7):1161. doi: 10.3390/foods14071161 (PMC11989002; doi:10.3390/foods14071161)
Supplement: Supplementary file 1 [file foods-14-01161-s001.zip › foods-3526887-supplementary.pdf]

Supplementary Table S1. Antibiotic susceptibility of *Schleiferilactobacillus harbinensis* WU01

Antibiotic disc	Result
Ampicillin (10 µg)	S
Vancomycin (30 µg)	R
Gentamicin (10 µg)	R
Erythromycin (15 µg)	S
Clindamycin (2 µg)	S
Tetracycline (30 µg)	S
Kanamycin (30 µg)	R
Chloramphenicol (30 µg)	S
Chloramphenicol (30 µg)	R

R, resistant; I, intermediate; S, susceptible

Supplementary Table S2. Predicted prophage regions in the genome of *S. harbinensis WU01*.

**Contig**	**Completeness**	**Score**	**Total proteins**	**Region Position**	**Most common phage**	**GC (%)**
NODE_5_length_339792_cov_121.440490	incomplete	20	20	325111-339790 info_outline	PHAGE_Lactob_phiAT3_NC_005893(3)	49.12%
NODE_9_length_21664_cov_106.810488	incomplete	130	25	282-21663 info_outline	PHAGE_Lactob_phiAT3_NC_005893(14)	46.64%
NODE_10_length_21087_cov_157.478344	intact	110	25	73-20896 info_outline	PHAGE_Lister_LP_101_NC_024387(10)	46.32%

Supplementary Table S3. The hydrogen bond interactions between Carnocin_CP52 and lipid molecules were analyzed from the 400–500 ns simulation

Acceptor	Donor	Occupancy (%)	Average distance (Å)	Average angle (degree)
PGR770@O33	Arg59@NH2	77.27	2.77	155.52
PGR740@O33	Arg65@NH2	55.45	2.76	158.96
PGR770@O33	Asn55@ND2	47.27	2.82	161.46
PC605@O33	Tyr61@OH	32.73	2.67	163.24
PC608@O33	His64@NE2	14.55	2.86	149.95
PC605@O33	Tyr75@OH	10.91	2.73	163.12

AOI	Start	End	Class
Contig_1	361,860	382,184	Carnocin_CP52

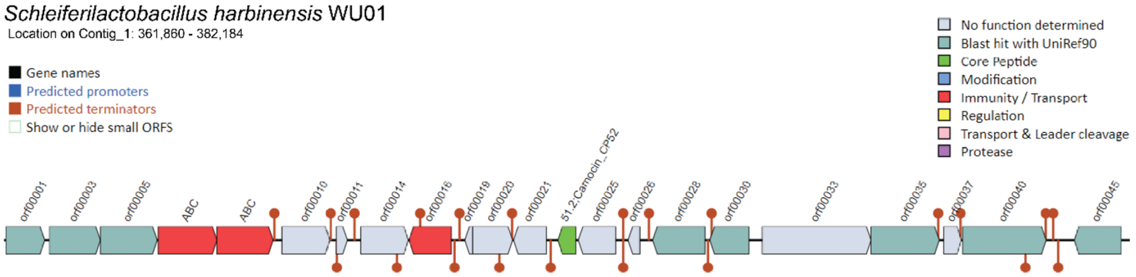

Supplementary Figure S1. *Schleiferilactobacillus harbinensis* WU01 genome analysis of bacteriocin genes arrangement using the BAGEL4 The area of interest at Contig_1 indicates Carnocin_CP52.

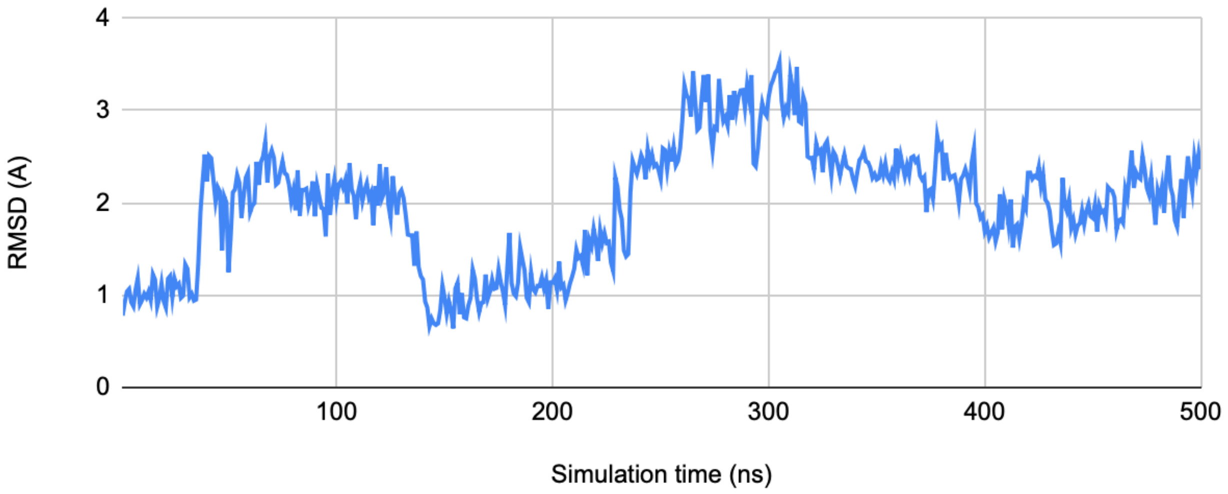

Supplementary Figure S2. The plots of root-mean-square displacement (RMSD) of Cα atoms of Carnocin_CP52 peptide

Supporting information

Amino acid sequence of Carnocin_CP52

NTKEKKLFQALDQAYMDLDVKKDPSLTSMIEENAKVLNASDSNDAYIHAVANLANGISR
YYLAHRGVPEVLMSIYQLIKADIPEAHVDADYYRQKAEAIGLSFFPVIFH
